# Biomechanical effects of deltoid muscle atrophy on rotator cuff tissue: a finite element study

**DOI:** 10.1038/s41598-024-67368-0

**Published:** 2024-07-30

**Authors:** Haiyan Wang, Lihua Chen, Guangming Xu, Hao Liu

**Affiliations:** 1 Department of Famous Traditional Chinese Medicine Hall, Shenzhen Bao’an Chinese Medicine Hospital, Shenzhen, Guangdong China; 2 Department of Rehabilitation, Shenzhen Bao’an Chinese Medicine Hospital, Shenzhen, Guangdong China; 3 Department of Orthopaedics, Shenzhen Hospital of Integrated Chinese and Western Medicine, Guangzhou University of Chinese Medicine, Shenzhen, Guangdong China; 4 Department of Chinese Medical Master Hall, Ruikang Hospital affiliated to Guangxi University of Chinese Medicine, Nanning, Guangxi China

**Keywords:** Muscle atrophy, Deltoid muscle, Rotator cuff, Stress, Diseases, Health care, Health occupations, Risk factors

## Abstract

The deltoid muscle and rotator cuff tissue are structural components that maintain the dynamic stability of the shoulder joint. However, atrophy of the deltoid muscle may affect the stability of the shoulder joint, which in turn alters the mechanical distribution of rotator cuff tissue. Currently, the effect of muscle volume changes in the deltoid muscle on reducing the load on the rotator cuff tissue is still unknown. Therefore, this paper intends to analyze the mechanical changes of rotator cuff tissue by deltoid muscle atrophy through finite elements. Based on previously published finite element shoulder models, the deltoid muscle was modeled by constructing deltoid muscle models with different degrees of atrophy as, 100% deltoid muscle (Group 1), 80% deltoid muscle (Group 2), and 50% deltoid muscle (Group 3), respectively. The three models were given the same external load to simulate glenohumeral joint abduction, and the stress changes in the rotator cuff tissue were analyzed and recorded. In all three models, the stress in the rotator cuff tissue showed different degrees of increase with the increase of abduction angle, especially in the supraspinatus muscle. At 90° of glenohumeral abduction, supraspinatus stress increased by 58% and 118% in Group 2 and Group 3, respectively, compared with Group 1; In the subscapularis, the stress in Group 3 increased by 59% and 25% compared with Group 1 and Group 2, respectively. In addition, the stress of the infraspinatus muscle and teres minor muscle in Group 2 and Group 3 were higher than that in Group 1 during the abduction angle from 30° to 90°. Deltoid atrophy alters the abduction movement pattern of the glenohumeral joint. During glenohumeral abduction activity, deltoid atrophy significantly increases the stress on the rotator cuff tissue, whereas normal deltoid volume helps maintain the mechanical balance of the rotator cuff tissue.

## Introduction

The deltoid muscle is an important stabilizing structure for shoulder movement^1^. However, deltoid muscle atrophy is often seen to varying degrees after rotator cuff injury or in elderly patients^2,3^. Deltoid muscle function and strength are essential for shoulder movement, and muscle strength and function are related to muscle volume^4,5^. Yoon et al.^5^ found that preoperative deltoid volume had a great influence on the actual results after reverse total shoulder arthroplasty. Strengthening exercises that increase the size of the deltoid muscles are known to improve clinical symptoms after rotator cuff injury^6^. However, due to the difficulty of measuring pressures within shoulder structures, there have been few studies on the effect of deltoid muscle volume changes on reducing rotator cuff tissue loading. Researchers have analyzed volumetric changes in shoulder muscles by measuring their cross-sectional area with MRI or ultrasound^7–9^. However, this does not effectively reflect the mechanical effects on rotator cuff tissue following deltoid muscle atrophy.

Recently, finite element (FE) analysis has been widely used in shoulder research. Inoue et al.^10^ explored the mechanics of supraspinatus injury by constructing 3D FE models of the rotator cuff muscles and the middle deltoid bundle. Researchers Construct 3D FE Models of Shoulder Muscles for Calculating Action Lines and Force Arms^11^. Yang et al.^12^ discussed the mechanical effects on shoulder joint tissues under different loading conditions. However, the above studies had their focus and did not explore the mechanical effects of deltoid muscle atrophy on rotator cuff tissue.

Therefore, we explored the mechanical effects of deltoid muscle atrophy on the shoulder tissues using an FE shoulder model. First, we modeled deltoid muscle atrophy by establishing different atrophy degrees. Then, we compared the differences in mechanical indexes of rotator cuff tissues in different models under external loads.

## Methods

### Established the shoulder model

**Figure 1:**
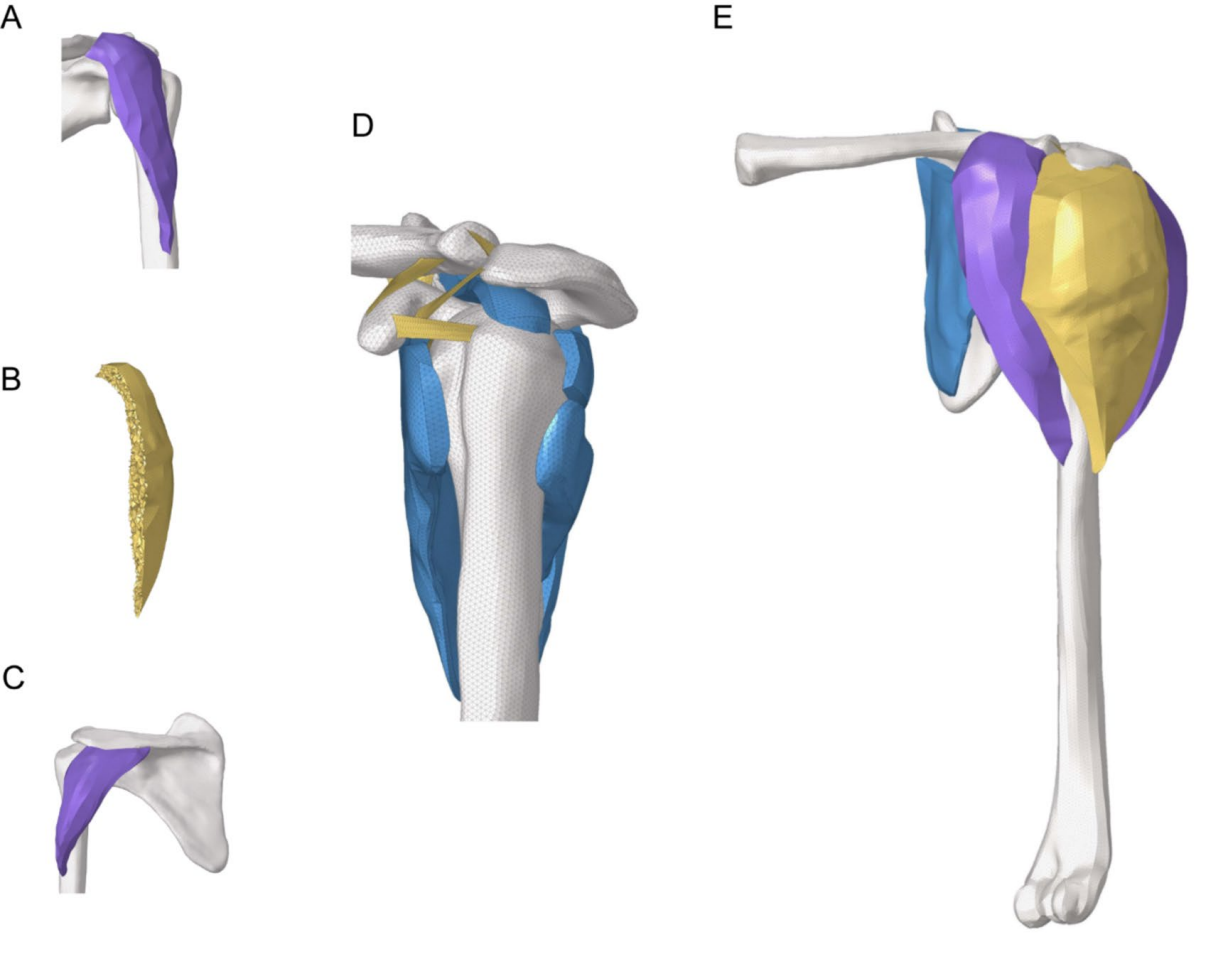
FE shoulder model. (**A**) Anterior deltoid bundle. (**B**) Internal view of the middle deltoid bundle. (**C**) Posterior deltoid bundle. (**D**) Overall view of rotator cuff. (**E**) The whole view of the FE shoulder model.

A shoulder model was constructed based on imaging data from healthy male subjects, as shown in Fig. 1. The model has been discussed in detail in a previous study^12,13^. The CT data in DICOM format was imported into Mimics modeling software and segmented and reconstructed. It was then imported into subsequent software for surface optimization, mesh segmentation, and formation of solid machine meshing. The model consisted of rotator cuff tissue, bony structures, and ligaments. Cancellous bone was modeled with solid elements. The rotator cuff consists of supraspinatus, infraspinatus, subscapularis and teres minor muscles. The deltoid muscle consists of the anterior, middle and posterior deltoids muslces. Rotator cuff tissue and deltoid muscle were defined as nonlinear hyperelastic and incompressible (Neo-Hooker) and modeled with solid elements. The acromioclavicular, glenohumeral and coracoclavicular ligaments were defined as nonlinear elasticity. The shoulder joint capsule was defined as isotropic hyperelasticity, which was modeled with shell element. The mesh size for each tissue was 1 mm. Detailed material parameters for each tissue are listed in Table 1^14–16^.

**Table 1:** Summary of material properties of the components.

Component	Material type	Material parameters
Scapula	Isotropic elastic	E = 15,000 MPa ν = 0.3
Clavicle		E = 15,000 MPa ν = 0.3
Humerus		E = 17,000 MPa ν = 0.3
Cancellous bone		E = 1000 MPa ν = 0.3
Rotator cuff tissue	Non-linear hyperelastic & incompressible (Neo-Hookean)	
Deltoid muscle		
Acromioclavicular ligament	Nonlinear elastic	E = 10.4 MPa ν = 0.3
Coracoclavicular ligament	Nonlinear elastic	E = 9.6 MPa ν = 0.3
Glenohumeral ligament	Nonlinear elastic	E = 150 MPa ν = 0.3

### Development of deltoid models with different degrees of atrophy

**Figure 2:**
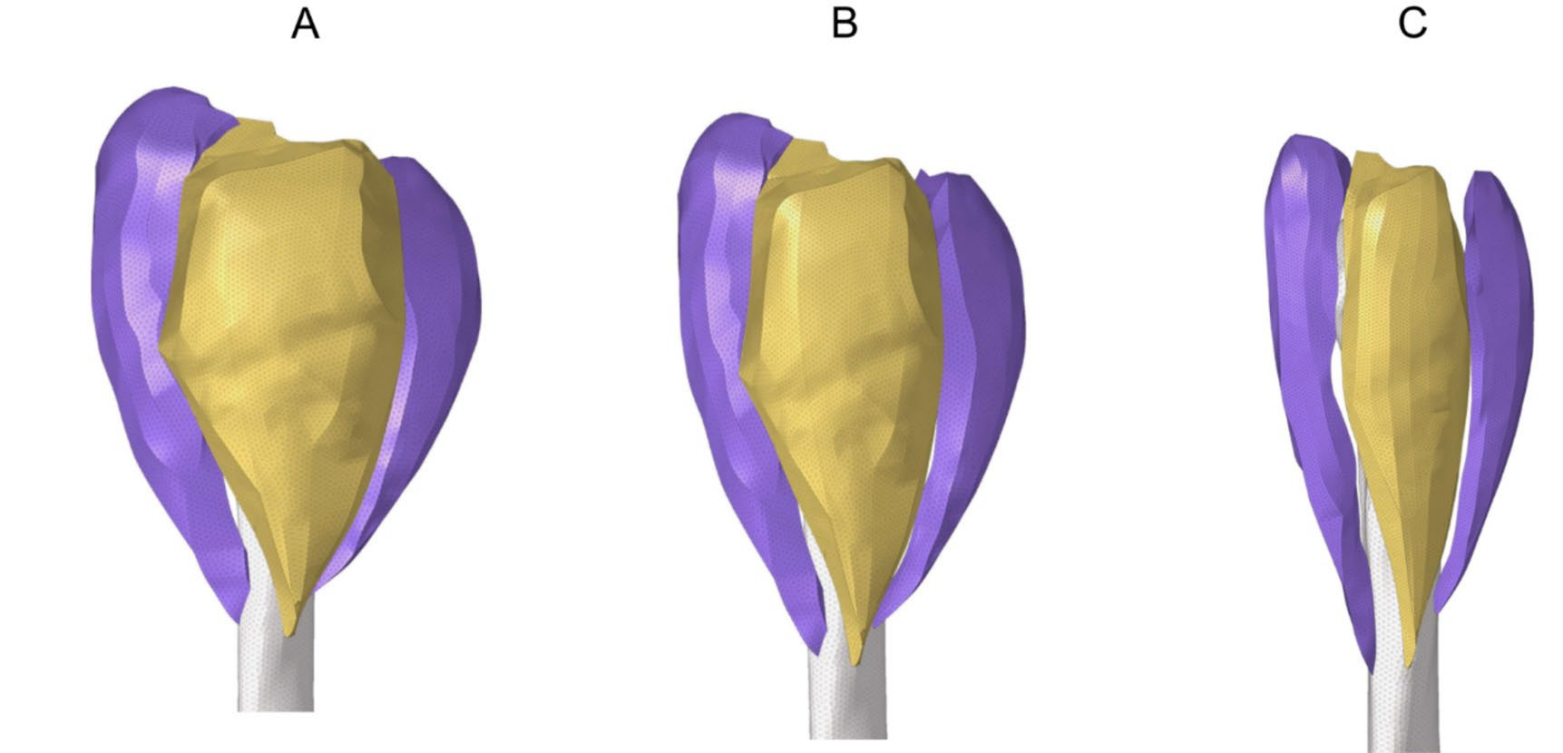
Deltoid muscle volumes: (**A**) 100%, (**B**) 80%, and (**C**) 50%.

To assess the effects of deltoid muscle volume changes on shoulder tissues, this study constructed deltoid muscle models with different degrees of atrophy by referring to previous literature^17^ as, 100% deltoid muscle (Group 1), 80% deltoid muscle (Group 2), and 50% deltoid muscle (Group 3) in Fig. 2, respectively. The contact between the deltoid muscle and the bone was set as bound.

### Loading conditions

In all FE models, loads were applied to the humerus and restricted the scapula’s degrees of freedom. A moment of 15 Nm was used to load the humerus beyond the abducted shoulder joint and gravity loading of the humerus was considered^18^. Stress changes in rotator cuff tissues at different angles in three models were recorded and analyzed to investigate the mechanical effects of different deltoid muscle volume changes on shoulder tissues.

## Results

### Stress in the supraspinatus muscle

**Figure 3:**
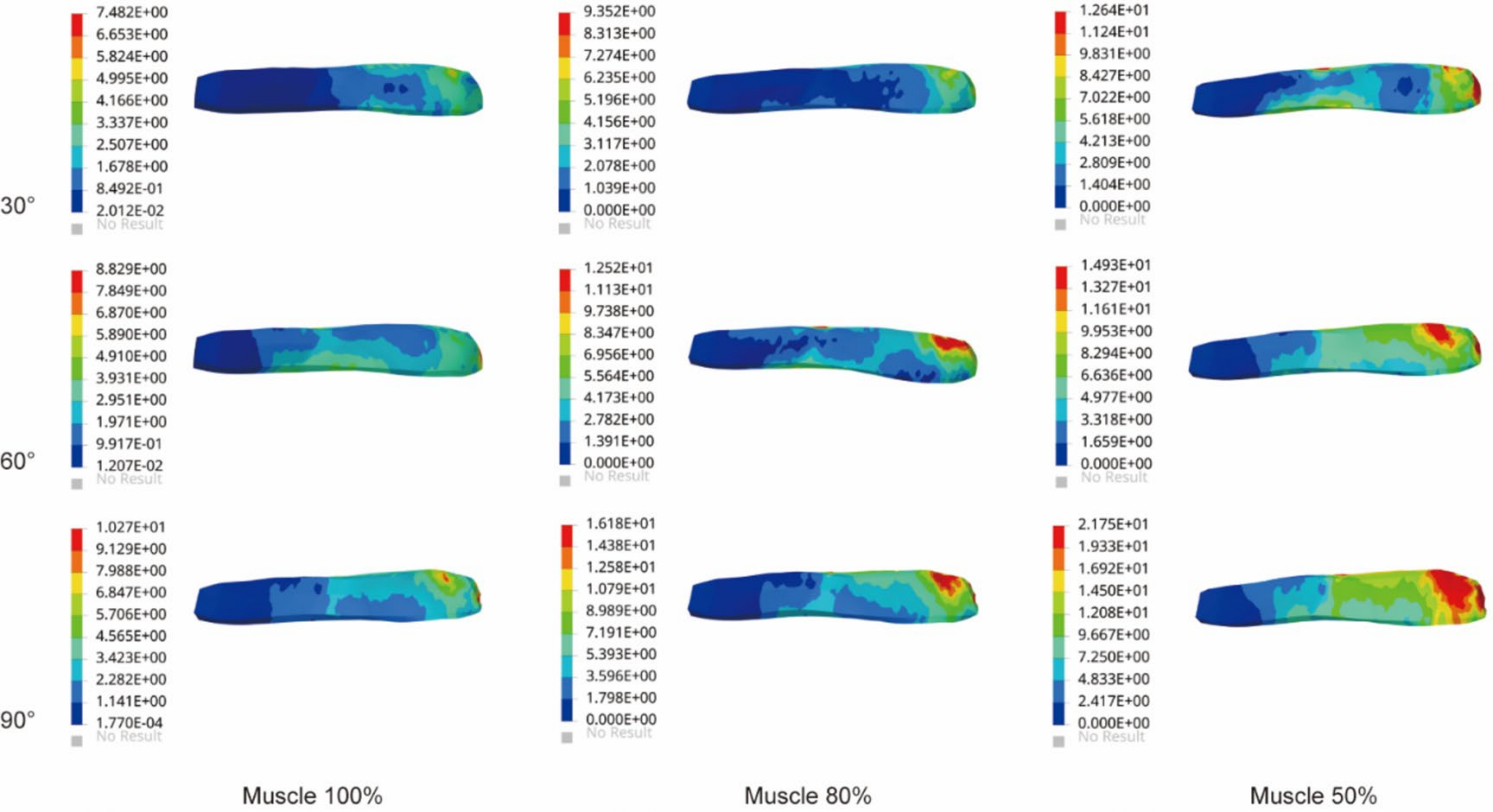
The maximum von Mises stress of supraspinatus muscle in three models.

The results in Fig. 3 showed stress changes in supraspinatus muscle. Stresses were mainly concentrated in the proximal part of the supraspinatus at its junction with the humerus. As the degree of atrophy and the angle of abduction increased, the region of high stress gradually increased. At 30° of abduction, the stresses of supraspinatus in the three groups were 7.48 MPa, 9.35 MPa, and 12.64 MPa. As the angle increased, the stresses of supraspinatus in the three groups reached a maximum of 90°, which were 10.27 MPa, 16.18 MPa, and 21.75 MPa, respectively. Compared with Group 1 at abduction 90°, the supraspinatus stresses in Groups 2 and 3 increased by 58% and 118%, respectively.

### Stress in the subscapularis muscle

**Figure 4:**
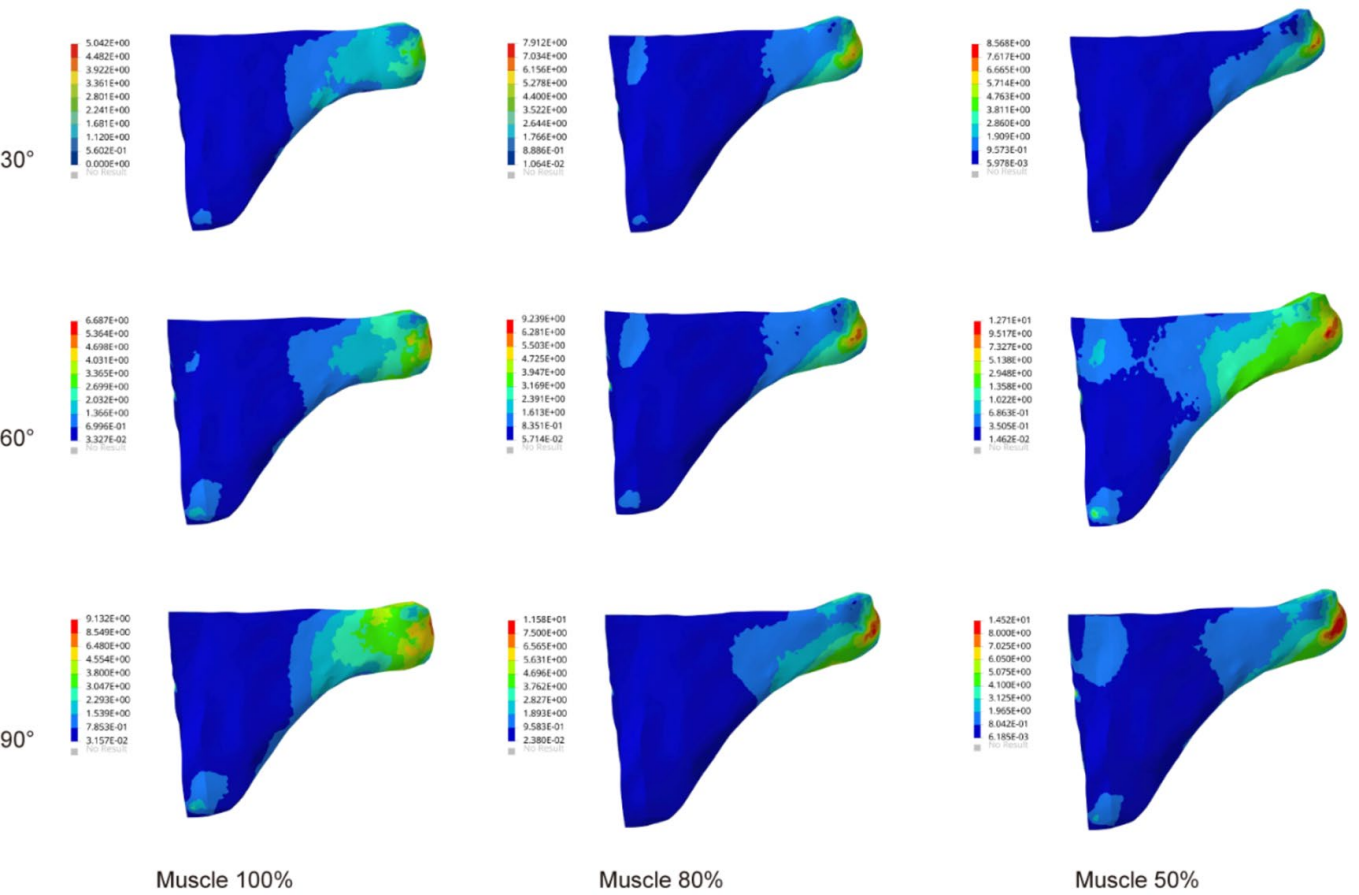
The maximum von Mises stress results in subscapularis muscle.

Figure 4 showed the stress changes of the subscapularis muscle in the glenohumeral abduction posture in the three groups of models. The stress cloud map of the subscapularis muscle showed significantly more red areas in group 3 than in groups 1 and 2 and was distributed in the junction with the humeral junction with higher stress values. With the increased angle, the stresses reached the maximum value at abduction 90°. The most significant increase in the stress value was 14.52 MPa in Group 3, which was a 59% increase compared to Group 1.

### Stress in the infraspinatus muscle

**Figure 5:**
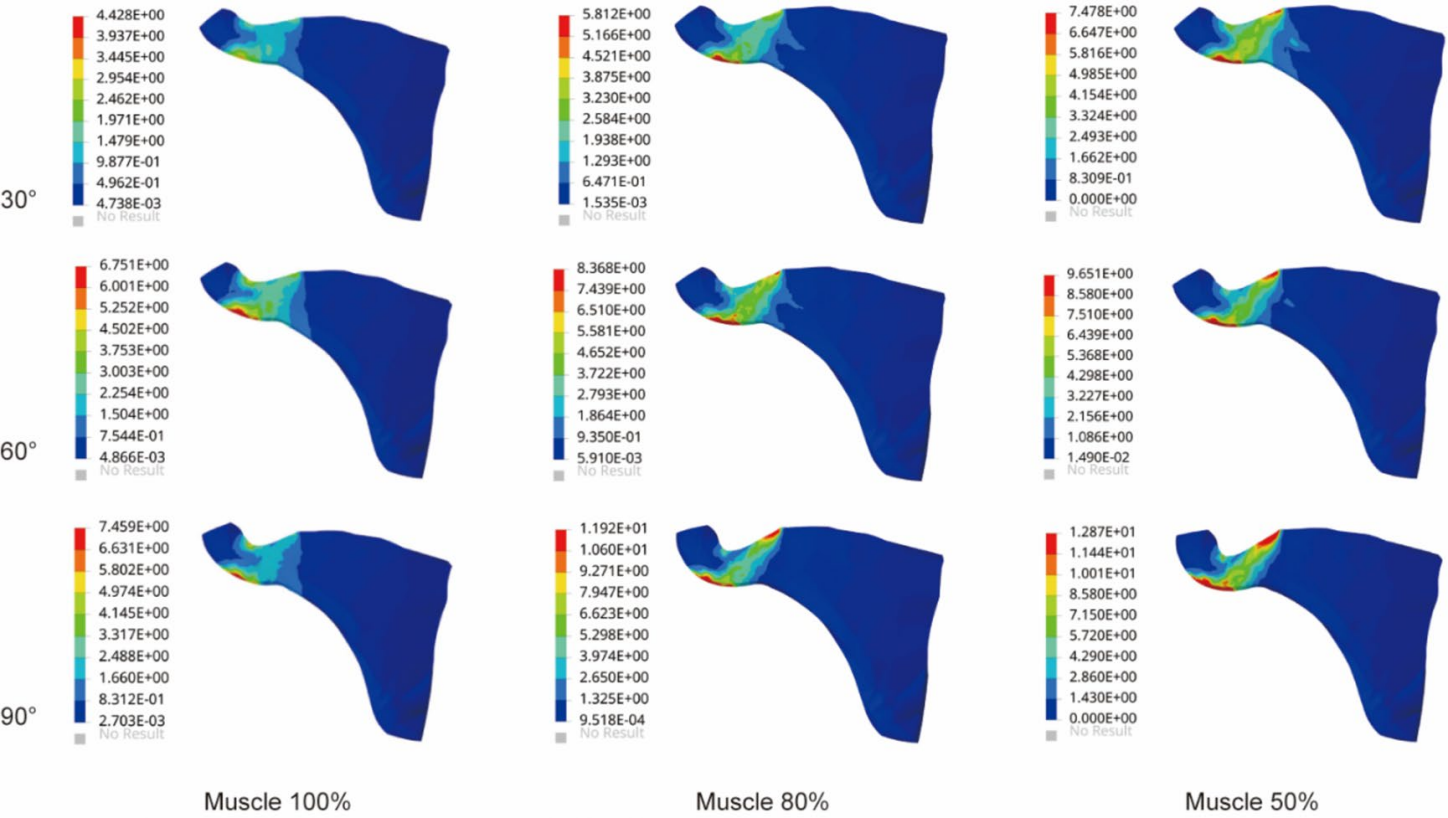
The maximum von Mises stress results in the infraspinatus muscle.

As shown in Fig. 5, the stress nephogram of infraspinatus muscle showed that the stress of infraspinatus muscle increased significantly with the increase of atrophy degree. The stress of the infraspinatus muscle was mainly concentrated around the junction with the proximal humerus. The change in the degree of atrophy resulted in a significant increase in stress. Compared with 30° of glenohumeral abduction, the stress in the infraspinatus muscle at abduction 60° increased by 52%, 44%, and 29% in the three groups. At 90° of glenohumeral abduction, the stress in Group 3 increased by 73% and 8% compared with Groups 1 and 2, respectively.

### Stress in the teres minor muscle

**Figure 6:**
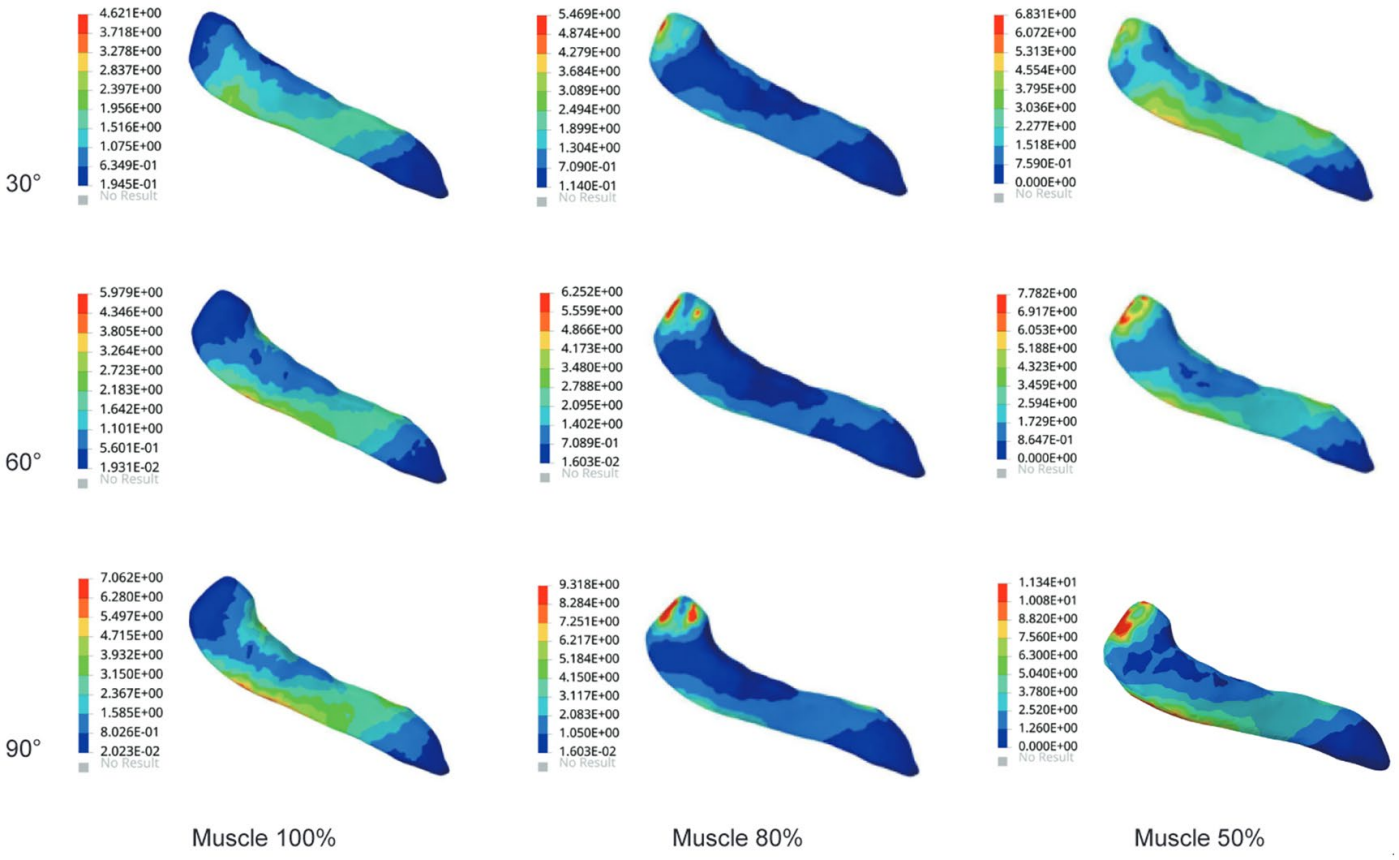
The maximum von Mises stress of the teres minor muscle.

The stress of teres minor muscle in all three groups in Fig. 6. Stress cloud maps of the teres minor muscle showed significant deformation and stress concentration at the junction of the teres minor and humerus as the angle increased. The angle increase and the deltoid muscle volume atrophy altered the stress growth trend of teres minor muscle. Compared with Group 1 at abduction 90°, the stress in the infraspinatus muscle increased by 32% in Group 2 and 61% in Group 3, respectively. The stress in the teres minor muscle was at a lower level compared to other rotator cuff tissues.

### Comparison of rotator cuff tissue stress

**Figure 7:**
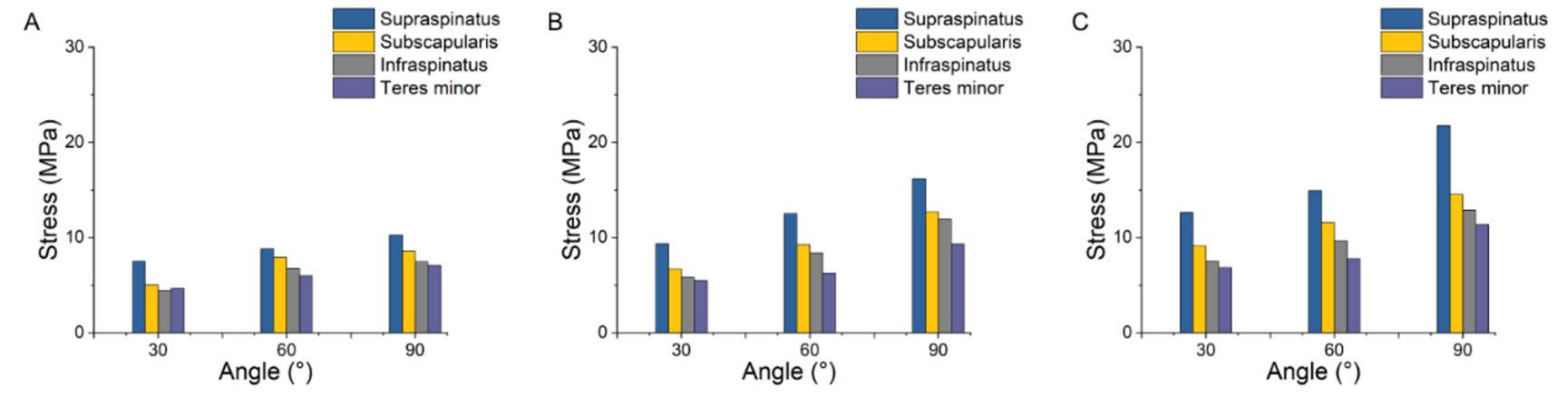
Comparison of stress in rotator cuff tissue during glenohumeral joint abduction. (**A**) 100% deltoid muscle volumes; (**B**) 80% deltoid muscle volumes; (**C**) 50% deltoid muscle volumes.

Figure 7 showed the stress in the rotator cuff tissue under glenohumeral joint abduction in different models. From 30° to 90° of abduction, the stress in the rotator cuff tissue increased to varying degrees. The supraspinatus was significantly more stressed than the other muscles. The stress value of the rotator cuff tissue was highest at 90° abduction. At 90° abduction, compared with subscapularis, infraspinatus, and teres minor, the stress in supraspinatus muscle of Group 1 increased by 20%, 38%, and 45%, respectively; the stress in supraspinatus muscle of Group 2 increased by 27%, 36%, and 74%; and the stress in supraspinatus muscle of Group 3 increased by 50%, 69%, and 92%.

## Discussion

The deltoid muscle is the abductor muscle of the shoulder joint and a structural component that maintains the dynamic stability of the glenohumeral joint^19^. Atrophy of the deltoid muscle affects the mechanical balance between the rotator cuff tissue and the shoulder joint. The anterior deltoid fascicle is not only involved in shoulder abduction during external rotation but also promotes internal rotation of the humeral head, while the posterior fibers are strongly involved in lateral extension and external rotation of the humeral head^20^. However, due to the physiological properties of the muscle, it is difficult to study the mechanical effects of deltoid atrophy on rotator cuff tissue by in vitro biomechanics. Instead, volume changes in the muscle are a common method for assessing changes in muscle force^20^. Therefore, we used FE methods to construct deltoid muscle models with different degrees of atrophy for subsequent analysis.

In this study, we observed that deltoid muscle atrophy leads to changes in the stress of the rotator cuff tissue. As shown in Figs. 3, 4, 5, 6 and 7, as the degree of deltoid muscle atrophy increased, the stress on the rotator cuff tissue appeared to increase to varying degrees during glenohumeral abduction. The most pronounced change in stress was in the supraspinatus, which was due to the muscle force during glenohumeral abduction being highly dependent on the action of the medial deltoid and supraspinatus muscles^21,22^. When the deltoid muscle atrophies, the supraspinatus muscle undergoes a significant change in its movement pattern increasing stress. In this article it is through passive simulation of shoulder abduction that the mechanical balance between the deltoid and rotator cuff tissues is disrupted due to the reduced muscle size, thus increasing the stress on the rotator cuff tissues. Since deltoid atrophy often occurs after rotator cuff injur^23–25^, and deltoid atrophy leads to an increase in stress on the rotator cuff tissues, the prevention of deltoid atrophy should be emphasized after rotator cuff injury.

Previous studies indicated that increasing the volume of the deltoid muscle significantly improves pain and function in rotator cuff injuries^6,26^. Our results show that normal deltoid muscle volume can effectively reduce the maximum stress in rotator cuff tissue and make the stress distribution more uniform, thereby improving the stress concentration in rotator cuff tissue. This could also explain biomechanically that increasing deltoid muscle volume through exercise improves symptoms of shoulder discomfort, which may reduce stress on the rotator cuff tissues and improve the mechanical balance of the shoulder tissues.

This study is based on computer-based FE analysis, which has some limitations. Firstly, the model is based on a single shoulder joint, which is unable to avoid the different results caused by the differences between individuals. Secondly, the superficial tissues and other muscles of the upper limb are not included in the model, which affects the shoulder movement and stress changes to a certain extent. Furthermore, the range of glenohumeral joint abduction angles studied in this paper is insufficient, and more angle and motion studies should be included in the future. Finally, the changes of deltoid atrophy and its effects on the shoulder joint in clinical practice are affected by many aspects, such as muscle atrophy is not necessarily isometric atrophy, which muscle atrophy is a multi-muscle group, and the activation of active muscles was not taken into account in the present study, so more clinical and in vitro studies are needed for further validation and analysis.

## Conclusion

In conclusion, this paper investigated the effect of deltoid muscle volume on rotator cuff tissue with different degrees of atrophy. The results showed that volumetric atrophy of the deltoid muscle can significantly alter the stress distribution in the rotator cuff tissue and lead to localized stress concentrations, particularly in the supraspinatus muscle, which may contribute to the risk of injury to the rotator cuff tissue.

## Data Availability

The original contributions presented in the study are included in the article/supplementary material, further inquiries can be directed to the corresponding authors.
